# Association Between Intraoperative Electroencephalogram Burst Suppression and Postoperative Delirium in Non-cardiac Surgery: A Systematic Review

**DOI:** 10.7759/cureus.110417

**Published:** 2026-06-07

**Authors:** Andrea Blanco Silva, Harumy Sarai Flores Calderon, Mercedes Camila Crespo Narváez, Mario Alfonso Blanco Gomez, Valeria Valentina Maldonado Rivera, Cristian Camilo Gil González, Juan Felipe Buitrago Navarro, Santiago Alejandro Chávez Fuenmayor, Tania Alejandra Suarez Gómez

**Affiliations:** 1 Anesthesiology and Critical Care Medicine, Centro Médico ABC, Ciudad de México, MEX; 2 Internal Medicine, Instituto Mexicano Del Seguro Social, Ciudad de México, MEX; 3 Medicine, Universidad de Cuenca, Cuenca, ECU; 4 Medicine, Fundacion Universitaria Juan N. Corpas, Bogotá, COL; 5 Medicine, Universidad del Cauca, Popayán, COL; 6 Medicine, Fundación Universitaria de Ciencias de la Salud, Bogotá, COL; 7 Medicine, Clínica Foscal, Floridablanca, COL; 8 Medicine, Universidad de las Americas, Quito, ECU; 9 Medicine, Fundación Universitaria Sanitas, Bogotá, COL

**Keywords:** burst suppression, electroencephalography, intraoperative electroencephalogram (eeg) burst, non-cardiac surgery, postoperative delirium

## Abstract

Postoperative delirium is a common complication in older surgical patients. Intraoperative EEG burst suppression may predict its occurrence. This study aimed to evaluate the association between burst suppression and postoperative delirium exclusively in non-cardiac surgery through systematic review and meta-analysis. A Preferred Reporting Items for Systematic Reviews and Meta-Analyses (PRISMA)-guided systematic review was conducted using PubMed, Cochrane Library, and ScienceDirect. Eligible studies included adult non-cardiac surgical patients assessing EEG burst suppression and postoperative delirium. Risk of bias was evaluated using RoB 2, the Newcastle-Ottawa Scale, and the Joanna Briggs Institute tools. Meta-analysis employed a random-effects Mantel-Haenszel model, with narrative synthesis for studies lacking extractable data. Across the included studies, the incidence of postoperative delirium (POD) ranged from 8.9% to 26%. Meta-analysis demonstrated that intraoperative burst suppression was significantly associated with POD occurrence (OR: 1.73, 95% CI: 1.03-2.90; I² = 38%). Patients with POD had significantly longer burst-suppression duration (mean difference of 25.31 min; p < 0.00001; I² = 0%). The pooled adjusted analysis confirmed an independent association between burst suppression and POD (OR: 2.69, 95% CI: 1.90-3.81; I² = 31%). Individual studies supported a dose-dependent relationship between the duration of suppression and the risk of delirium. Anesthetic agents influenced the suppression burden - propofol was associated with longer durations, whereas desflurane was associated with increased POD risk, independent of suppression. Preoperative cognitive impairment and frailty were identified as significant effect modifiers, increasing susceptibility to burst suppression and subsequent delirium. Intraoperative EEG burst suppression is significantly associated with postoperative delirium in non-cardiac surgery. Duration and patient vulnerability influence risk, highlighting the potential of duration as a modifiable intraoperative predictor.

## Introduction and background

The most frequent major surgical consequence in older persons is postoperative delirium (POD), which is linked to longer hospital stays, more ICU admissions, postoperative neurocognitive disorders, and death [1,2]. It manifests as a neuropsychiatric consequence after surgery and is a complex organic brain condition. POD presents a significant issue in perioperative treatment, characterized by sudden changes in awareness, attention, and cognition [3,4]. Increased patient morbidity, extended hospital stays, and an increased risk of long-term cognitive loss, particularly in elderly patients, are some of its consequences [5].

Given the organic causes of postoperative delirium, electroencephalography (EEG) monitoring of brain activity after surgery appears to be a useful method for anticipating this problem [6]. As a result, even though there is little high-quality data to support this approach, new European recommendations on postoperative delirium care encourage intraoperative monitoring of anesthetic depth and EEG patterns, particularly the burst-suppression pattern [7]. Burst suppression is a distinct EEG pattern characterized by alternating bursts of high-voltage electrical activity and periods of near-complete cortical silence, reflecting profound suppression of cerebral metabolic activity and neuronal synchrony. It is most commonly observed during deep anesthesia and has been associated with neuronal injury and dysregulation of neurotransmitter systems implicated in the pathogenesis of delirium. Importantly, burst suppression is a potentially modifiable intraoperative exposure; anesthetic depth and agent selection directly influence its occurrence, meaning that clinicians who identify this pattern in real time may be able to adjust management to reduce the risk of delirium.

Intraoperative electroencephalogram (EEG) burst suppression has emerged as a candidate neurophysiological marker linked to POD development, and several systematic reviews have explored this relationship. Intraoperative electroencephalogram (EEG) burst suppression has emerged as a candidate neurophysiological marker linked to POD development. Park et al. and Likhvantsev et al. both conducted meta-analyses confirming a statistically significant association between burst suppression and POD, reporting pooled odds ratios of 1.492 and a 41% increase in relative risk [8,9]. However, neither review stratified analyses by surgery type, meaning their pooled estimates include cardiac surgical patients - a population with fundamentally distinct pathophysiological mechanisms for delirium, including cardiopulmonary bypass-related cerebral hypoperfusion, microemboli, and systemic inflammatory cascades absent in non-cardiac surgery - thereby introducing substantial heterogeneity that may confound the estimated association.

Two additional reviews by Sumner et al. and Sun et al. evaluated processed EEG-guided anesthetic delivery rather than burst suppression as a discrete electrographic exposure, further incorporating ICU sedation contexts and cognitive rather than clinically diagnosed POD endpoints, limiting their perioperative applicability [10,11]. To date, no systematic review has specifically examined intraoperative EEG burst suppression and POD exclusively in non-cardiac surgical patients. Given that non-cardiac surgery represents the vast majority of the global operative burden, and that burst suppression-related neurotoxicity mechanisms may differ meaningfully from those in cardiac surgery, the present systematic review and meta-analysis address this evidence gap by synthesizing data exclusively from non-cardiac surgical populations, with burst suppression as the defined EEG exposure and clinically diagnosed POD as the primary outcome.

## Review

Methods

Study Design and Reporting Framework

This systematic review and meta-analysis were conducted and reported in accordance with the Preferred Reporting Items for Systematic Reviews and Meta-Analyses (PRISMA) 2020 guidelines [12]. The study protocol was not prospectively registered on the International Prospective Register of Systematic Reviews. This review was not prospectively registered on the International Prospective Register of Systematic Reviews (PROSPERO) prior to data extraction, which is acknowledged as a limitation; subgroup analyses described below should therefore be interpreted as exploratory rather than strictly confirmatory. No ethical approval was required, as this study involved secondary analysis of previously published data.

Inclusion Criteria

Studies were considered eligible if they included adult patients aged 18 years or older, with particular emphasis on elderly populations, undergoing non-cardiac surgery under general anesthesia. The exposure of interest was intraoperative EEG burst suppression or related suppression metrics, including suppression duration or burst suppression ratio. Studies were required to include a comparator group comprising patients without burst suppression or with lower levels of suppression. The primary outcome had to be postoperative delirium assessed using validated tools such as the Confusion Assessment Method (CAM), CAM-ICU, or other standardized clinical diagnostic criteria. Eligible study designs included randomized controlled trials, prospective cohort studies, retrospective cohort studies, and observational studies. Only studies published in English were included.

Exclusion Criteria

Studies were excluded if they involved cardiac or neurosurgical populations or pediatric patients, or were designed as case reports, case series, editorials, or review articles. Additionally, studies lacking a clear assessment of postoperative delirium or without defined EEG suppression measurements were excluded from the analysis.

Information Sources and Search Strategy

A comprehensive and systematic literature search was performed across multiple electronic databases, including PubMed/MEDLINE, Cochrane Library, and ScienceDirect. The search encompassed all studies from database inception to the most recent date prior to analysis. The search strategy incorporated both Medical Subject Headings (MeSH) terms and free-text keywords related to “burst suppression,” “electroencephalogram” or “EEG,” “postoperative delirium” or “POD,” and “non-cardiac surgery.” Boolean operators such as AND and OR were applied to refine the search and enhance both sensitivity and specificity. In addition to database searches, the reference lists of all included studies were manually screened to identify any additional relevant articles that may have been missed.

The following search strategies were used in PubMed (EEG OR electroencephalography) AND ("burst suppression" OR "EEG suppression") AND (delirium OR "acute confusional state") AND (surgery OR intraoperative), ScienceDirect (EEG OR electroencephalography) AND ("burst suppression" OR "EEG suppression") AND (delirium OR "acute confusional state") AND (surgery OR intraoperative), and the Cochrane Library (electroencephalogram OR EEG) AND ("burst suppression" OR "EEG suppression" OR "burst suppression ratio") AND (delirium OR "postoperative delirium") AND (intraoperative OR postoperative) AND ("non-cardiac surgery" OR surgery).

Study Selection Process

All retrieved records were imported into reference management software, where duplicate entries were identified and removed. Two independent reviewers conducted an initial screening of titles and abstracts to assess relevance, followed by a full-text review of potentially eligible studies based on the predefined inclusion and exclusion criteria. Any discrepancies between reviewers were resolved through discussion, and when necessary, consultation with a third reviewer. The overall study selection process was documented and presented using a PRISMA flow diagram.

Data Extraction

Data were extracted using a standardized data collection form to ensure consistency and completeness. Extracted variables included study characteristics such as author name, publication year, and study design; population details, including sample size, mean age, and type of surgery; EEG-related parameters, such as presence, duration, or proportion of burst suppression; and methods used for postoperative delirium assessment. Additionally, data on the incidence of POD, reported effect estimates, including odds ratios, relative risks, and beta coefficients, along with their corresponding confidence intervals, and key findings and statistical significance were collected. Data extraction was performed independently by two reviewers to minimize errors and discrepancies.

Risk of Bias Assessment

Risk of bias was assessed independently by two reviewers using validated, study-design-specific tools. Disagreements were resolved by consensus or senior author adjudication. For randomized controlled trials, the Cochrane Risk of Bias 2 (RoB 2) tool was applied, evaluating the following five domains: (1) randomization process, (2) deviations from intended interventions, (3) missing outcome data, (4) outcome measurement, and (5) selection of reported results. For observational studies (prospective and retrospective cohorts, cross-sectional studies), the Newcastle-Ottawa Scale (NOS) was applied across the following three domains: selection of study groups, comparability of groups, and ascertainment of exposure and outcome. Studies scoring 7-9 stars were classified as low risk of bias; 4-6 stars as moderate; and 0-3 stars as high risk. Risk-of-bias results are presented both narratively and graphically using RoB 2 traffic-light plots and NOS scoring tables. For a cross-sectional study, the Joanna Briggs Institute (JBI) was used. This tool was applied to evaluate methodological quality, including selection criteria, measurement validity, and control of confounding factors.

Data Synthesis and Statistical Analysis

Data synthesis was structured around predefined outcome domains, including the overall incidence of postoperative delirium (POD), the association between intraoperative burst suppression and POD, the relationship between burst suppression duration and POD, and the pooled independent effect of burst suppression on the risk of delirium. Quantitative meta-analyses were performed, where appropriate, using a random-effects Mantel-Haenszel model to account for inter-study variability, with odds ratios (ORs) and 95% confidence intervals (CIs) used for dichotomous outcomes and pooled mean differences for continuous variables such as suppression duration. Meta-analyses were conducted using Review Manager (RevMan version 5.4; Copenhagen, Denmark: Cochrane Collaboration). Separate meta-analyses were conducted to evaluate the incidence of burst suppression in POD-positive vs. POD-negative patients, its duration in relation to POD development, and its independent association with delirium after adjustment for confounders. It is acknowledged that three separate meta-analyses were performed on overlapping study sets; readers should interpret overall inferences accordingly. The pooled adjusted odds ratio (section 3.6) combines observational studies that adjusted for different confounders using different models, and this estimate should be interpreted with appropriate caution. The pooled mean difference for burst suppression duration (section 3.5) is based on a small number of studies; the resulting I² of 0% should not be interpreted as confirming homogeneity, given the limited sample size.

For studies in which extractable data were not reported with sufficient detail to permit inclusion in quantitative synthesis, a structured narrative synthesis was performed, and findings were presented as individual study evidence to ensure comprehensive inclusion of all relevant results. In addition to pooled analyses, qualitative synthesis was used to integrate findings related to anesthetic agent effects, EEG-guided anesthetic titration strategies, and preoperative vulnerability factors such as cognitive impairment and frailty. Subgroup analyses were prespecified and planned a priori in the protocol; however, given the absence of prospective PROSPERO registration, they should be considered exploratory based on surgical type (e.g., spine vs. mixed non-cardiac procedures), EEG measurement methodology (visual interpretation vs. device-derived indices), and patient characteristics, including age and baseline cognitive status. Planned subgroup analyses included surgical type (e.g., spine vs. mixed non-cardiac procedures), EEG measurement methodology (visual interpretation vs. device-derived indices), and patient characteristics, including age and baseline cognitive status. Due to insufficient data across studies, formal subgroup analyses could not be performed, and findings are reported narratively. Assessment of publication bias using funnel plots and Egger’s regression test was not performed due to the limited number of studies included in each meta-analysis, which was insufficient to reliably evaluate small-study effects.

Results

**Figure 1:**
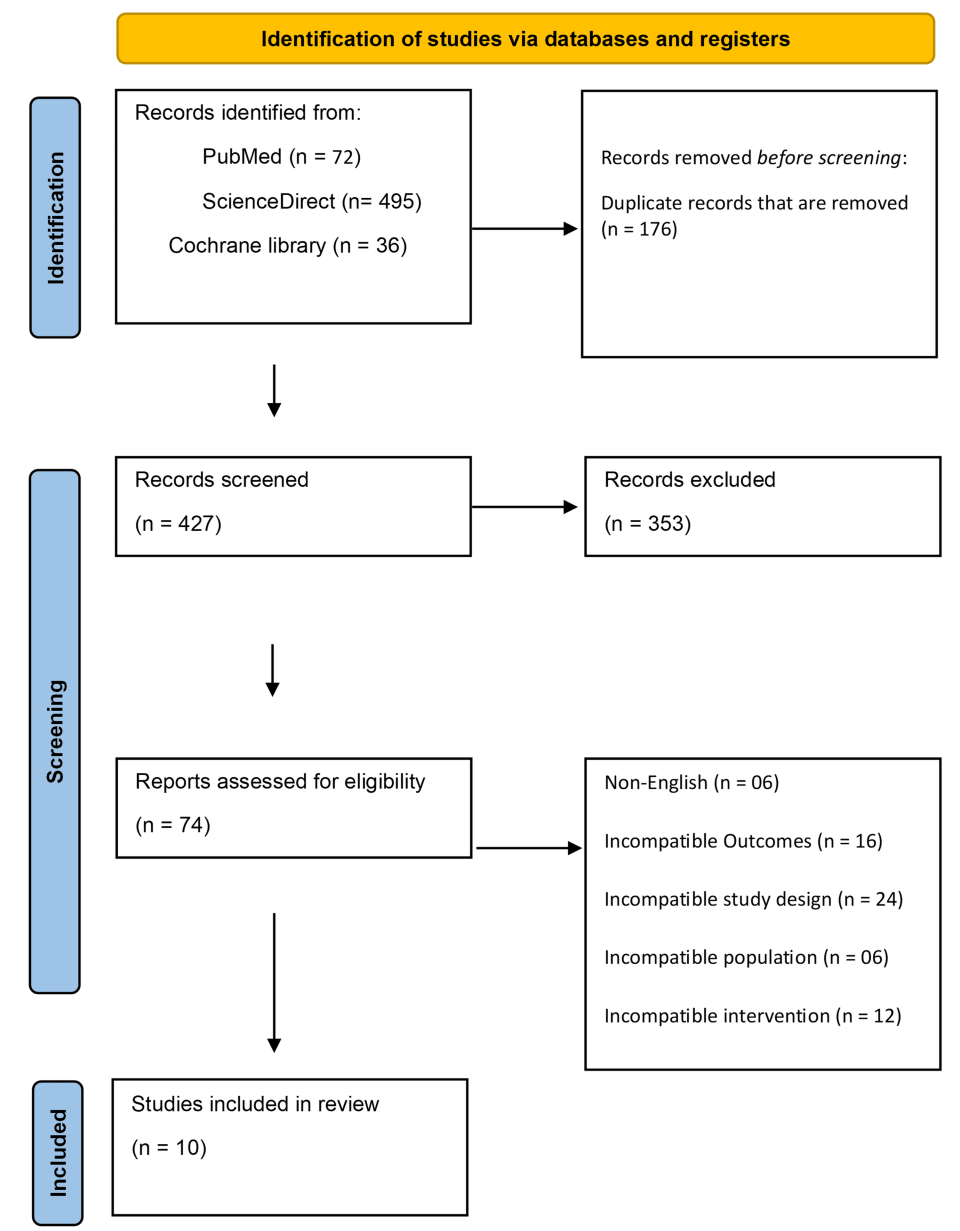
PRISMA flow chart. PRISMA: Preferred Reporting Items for Systematic Reviews and Meta-Analyses

The study selection process is illustrated in the PRISMA flow diagram (Figure 1). A total of 603 records were identified through database searching. After removal of 176 duplicates, 427 records remained for title and abstract screening, of which 353 were excluded for not meeting the inclusion criteria. The full texts of 74 articles were assessed for eligibility, resulting in the exclusion of 64 studies for reasons including an inappropriate study population (cardiac/neurosurgical/ICU settings), lack of discrete reporting of burst suppression, or absence of relevant outcomes. Ultimately, 10 studies fulfilled all eligibility criteria and were included in the qualitative and quantitative synthesis. Characteristics of the studies included are summarized in Table 1.

**Table 1:** Characteristics of the studies included. PANDA-G study: Perioperative Neurocognitive Disorders and Anesthesia-Geriatric study; POD: postoperative delirium; BS: burst suppression; CAM: Confusion Assessment Method; PODE: Postoperative Delirium in Elderly Patients; SuDoCo study: Surgery, Delirium and Cognitive Outcomes study; TIVA: total intravenous anesthesia; PACU: post-anesthesia care unit; RASS: Richmond Agitation-Sedation Scale; ADAPT-2: Anesthetic Depth and Postoperative Delirium Trial-2

Studies	Year	Study design	Population type and number	Mean age, years	Surgical procedure	POD assessment method	EEG pattern	POD positive and negative numbers
Ren et al. [13]	2023	Prospective cohort study	Elderly patients > 60 years undergoing lumbar spine surgery; n = 90	All >60 years	Lumbar internal fixation surgery	CAM	Burst suppression vs. non-burst suppression	POD incidence higher in BS group
Reese et al. [14]	2023	Prospective cohort study (secondary analysis of 2 cohorts)	Older adults ≥60 years undergoing non-cardiac, non-neurologic surgery; n = 83	68	Mixed non-cardiac, non-neurologic surgeries (≥ 2-h duration)	Standard delirium assessment	Burst suppression and pre-burst suppression (reduced EEG power preceding BS)	Exact POD counts not reported; ~20.5% had at least one BS episode
Pawar et al. [15]	2026	Retrospective cohort study (secondary analysis of PANDA-G study)	Older adults undergoing spine surgery; n = 239	Older adults	Spine surgery	Standard delirium assessment (not specified, but within PANDA-G protocol)	Burst suppression (visual EEG analysis vs. device-derived ratio)	BS present in 73.45% of delirium patients vs. 50.9% in non-delirium patients
Fritz et al. [16]	2016	Observational cohort study	Adult patients undergoing surgery with planned ICU admission; n = 727 (619 assessed for POD)	62 ± 14	Mixed major surgeries requiring ICU admission (non-cardiac dominant but not exclusively specified)	CAM-ICU assessed twice daily	EEG suppression (duration-based analysis)	POD positive = 162 (26%); POD negative = 457 (74%)
Tang et al. (ADAPT-2 Trial) [17]	2020	Randomized controlled trial	Elderly patients ≥65 years undergoing elective non-cardiac surgery; n = 204	72 ± 5	Elective major non-cardiac surgery	Standard delirium assessment (within trial protocol, first 3 postoperative days)	EEG suppression duration (percentage of surgical time)	POD incidence: intervention = 17%; control = 20%
Jung et al. [18]	2021	Prospective cross-sectional observational study	Adult patients undergoing elective abdominal surgery; n = 80	70.68	Elective abdominal surgery under general anesthesia (sevoflurane + sufentanil)	Not explicitly specified (clinical delirium diagnosis of PODE)	EEG burst suppression and suppression duration	POD positive = 13 (16.25%); POD negative = 67 (83.75%)
Koch et al. [19]	2023	Secondary analysis of prospective cohort (SuDoCo trial)	Elderly patients >60 years undergoing general anesthesia; n = 1,277	69.7	Mixed non-cardiac surgeries under general anesthesia	Standard delirium assessment (within trial protocol)	Burst suppression duration (minutes)	POD incidence = 18.7% (~239 patients); no POD ≈ 81.3% (~1,038 patients)
Lele et al. [20]	2022	Retrospective observational quality improvement study	Adult patients undergoing spinal instrumentation surgery; n = 112	Age range 20-88 years	Spinal instrumentation surgery under TIVA	Not explicitly specified (clinical diagnosis of postoperative delirium)	Burst suppression (presence, duration, and proportion of monitoring time)	POD positive = 10 (8.9%); POD negative = 102 (91.1%)
Hesse et al. [21]	2019	Prospective observational study	Adult patients undergoing general anesthesia for non-neurologic procedures; n = 626	56	Mixed non-cardiac surgeries	PACU delirium assessment (standard clinical methods)	Burst suppression and EEG emergence patterns (spindle activity, trajectory analysis)	POD (PACU delirium) positive = 125 (20%); POD negative = 501 (80%)
Lutz et al. [22]	2022	Prospective observational study	Adult patients undergoing general anesthesia; analyzed cohort n = 116 (from 169 eligible)	PACU-D no: 59 years. PACU-D yes: 70 years	Mixed non-cardiac surgeries under general anesthesia	CAM-ICU and RASS used for PACU delirium assessment	EEG alpha power, coherence, and spectral patterns	POD (PACU delirium) positive = 25; POD negative = 91

Study Characteristics

The systematic search yielded 10 studies that fulfilled the predefined inclusion criteria, collectively enrolling 3,574 patients undergoing non-cardiac surgery under general anesthesia. Included study designs comprised prospective cohort studies [13,14,21,22], one randomized controlled trial [17], retrospective observational cohort studies [15,20], an observational cohort study [16], a cross-sectional study, and a secondary analysis of a prospective cohort [18,19]. Sample sizes ranged from 80 to 1,277 patients.

The majority of included studies focused on elderly populations, with six studies enrolling patients aged 60 years or older [13-15,17-19]. Mean ages ranged from 56 to 72 years [17,21]. Lele et al. enrolled patients across a broad age range of 20-88 years [20]. Surgical procedures were heterogeneous but uniformly non-cardiac, encompassing lumbar spine and spinal instrumentation surgery [13,15,20], elective abdominal surgery [18], mixed major surgeries [16], and mixed non-cardiac elective surgeries [14,17,19,21,22].

Postoperative delirium was assessed using validated instruments across most studies. The Confusion Assessment Method (CAM) was employed by Ren et al. [13], the CAM-ICU by Fritz et al. [16], and the combined CAM-ICU with Richmond Agitation-Sedation Scale (RASS) by Lutz et al. [22]. Two studies applied standard PACU-based clinical delirium assessment protocols [20,21], while three studies used structured trial-protocol-driven assessments [15,17,19]. Jung et al. reported a clinical diagnosis of delirium without specifying a formal, validated instrument [18].

Quality Assessment

**Figure 2:**
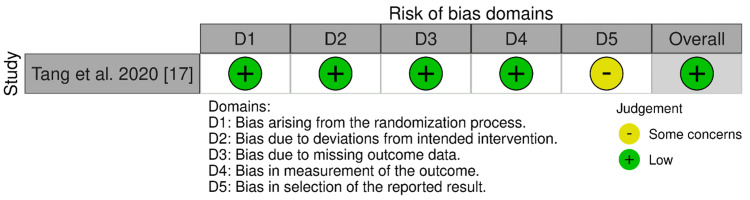
Quality assessment of RCT by RoB 2 tool. RoB 2: Cochrane Risk of Bias 2; RCT: randomized controlled trial

Most cohort studies were of moderate to high methodological quality based on the Newcastle-Ottawa Scale, with several large prospective cohorts achieving the highest scores. The methodological quality of the included cohort studies is summarized in Table 2. The randomized controlled trial demonstrated a low overall risk of bias using the RoB 2 tool [17]. The risk of bias for the randomized controlled trial is illustrated in Figure 2. The single cross-sectional study was rated as moderate quality using the JBI checklist, primarily due to limited control of confounding factors [18]. The quality assessment of the cross-sectional study is detailed in Table 3.

**Table 2:** NOS scale for quality assessment of cohort studies. Studies scoring 7-9 stars were classified as low risk of bias; 4-6 stars as moderate; and 0-3 stars as high risk. NOS: Newcastle-Ottawa Scale

Studies	Selection (4)	Comparability (2)	Outcome (3)	Total (9)	Quality
Ren et al. (2023) [13]	★★★★	★	★★	7/9	Good
Reese et al. (2023) [14]	★★★	★	★★	6/9	Moderate
Pawar et al. (2026) [15]	★★★	★	★★	6/9	Moderate
Fritz et al. (2016) [16]	★★★★	★★	★★★	9/9	High
Koch et al. (2023) [19]	★★★★	★★	★★★	9/9	High
Lele et al. (2022) [20]	★★★	★	★★	6/9	Moderate
Hesse et al. (2019) [21]	★★★★	★★	★★★	9/9	High
Lutz et al. (2022) [22]	★★★★	★★	★★★	9/9	High

**Table 3:** Quality assessment of cross-sectional study by JBI-checklist. JBI: Joanna Briggs Institute; POD: postoperative delirium

Domain	Judgment
Inclusion criteria clearly defined	Yes
Study subjects and setting described	Yes
Exposure measured validly (EEG)	Yes
Outcome measured reliably (POD)	Yes (though tool not specified)
Confounding factors identified	Partial
Strategies to deal with confounders	Partial
Statistical analysis appropriate	Yes

Overall Incidence of Postoperative Delirium

The overall incidence of POD varied considerably across the included studies, reflecting differences in patient demographics, surgical complexity, anesthetic agents, and the timing and methodology of delirium assessment. Fritz et al. recorded the highest POD incidence, with 162 of 619 assessed patients (26%) fulfilling delirium criteria [16]. Hesse et al. identified PACU delirium in 125 of 626 patients (20%), a figure closely mirrored by Lutz et al., who reported 25 of 116 patients (21.5%) with PACU delirium [21,22]. Koch et al., drawing on the largest included cohort, documented a POD incidence of 18.7% (~239 of 1,277 patients) [19]. Tang et al. reported rates of 17% and 20% in the intervention and control arms, respectively [17], while Jung et al. identified POD in 13 of 80 patients (16.25%) [18]. Lele et al. recorded the lowest incidence at 8.9% (10 of 112 patients) [20].

Association Between the Presence and Incidence of Intraoperative Burst Suppression and Postoperative Delirium

**Figure 3:**
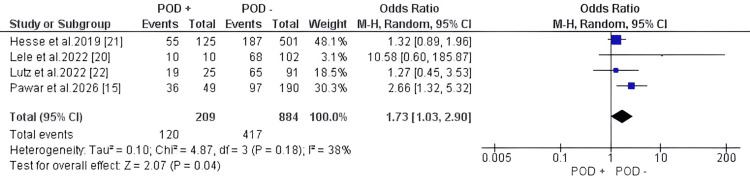
Forest plot of association of incidence of burst suppression with POD. POD: postoperative delirium

To quantify the association between burst suppression occurrence and POD, a meta-analysis was performed pooling data from four studies that reported the incidence of burst suppression in both POD-positive and POD-negative patients. Using a random-effects Mantel-Haenszel model, the pooled odds ratio for burst suppression occurrence in POD-positive patients relative to POD-negative patients was 1.73 (95% CI: 1.03-2.90), with a test for overall effect of Z = 2.07 (p = 0.04), confirming that intraoperative burst suppression was significantly more likely to occur in patients who subsequently developed POD. Heterogeneity was moderate (tau² = 0.10; chi² = 4.87, df = 3, p = 0.18; I² = 38%). Figure 3 illustrates the forest plot of the meta-analysis assessing the association between the incidence of intraoperative burst suppression and postoperative delirium (POD), demonstrating a statistically significant increased risk in POD-positive patients.

Individual Study Evidence

Beyond the meta-analytic pooled estimate, individual study data further corroborated the association between burst suppression presence and POD. Pawar et al. demonstrated that burst suppression was present in 73.45% of delirium patients vs. 50.9% of non-delirium patients (p = 0.001), and this association was a stronger predictor of POD than age alone (β = 0.182, p = 0.002 vs. β = 0.009, p = 0.082) [15]. Pawar et al. also identified potential measurement discrepancies between visually scored and device-derived burst suppression ratios, suggesting that automated EEG-derived metrics may systematically underestimate suppression in some operative contexts [15]. Burst suppression specifically during the anesthetic maintenance phase was also significantly more common in delirium patients (67.2% vs. 46.3%, p = 0.004). Ren et al. confirmed this in elderly patients undergoing lumbar spine surgery, reporting an adjusted OR of 4.954 (95% CI: 1.034-23.736, p = 0.045) for POD in patients who experienced intraoperative burst suppression, with frailty significantly more prevalent in the burst suppression group (p = 0.048) and the hyperactive subtype predominating among delirium cases [13]. Reese et al. demonstrated that the percentage of total surgical time spent in burst suppression was significantly correlated with POD incidence (OR: 1.34, 95% CI: 1.01-1.78, p = 0.043) in older adults undergoing non-cardiac, non-neurologic surgeries lasting at least two hours [14]. Lutz et al. importantly demonstrated that the timing of burst suppression within the anesthetic course was critical - suppression during the maintenance phase was significantly associated with PACU delirium (52% vs. 20.9%; p = 0.002), whereas overall suppression prevalence across the entire anesthetic course (75% vs. 71.4%; p = 0.651) and induction-phase suppression specifically (48% vs. 61.5%; p = 0.223) did not reach statistical significance [22].

Association Between Burst Suppression Duration and Postoperative Delirium

**Figure 4:**
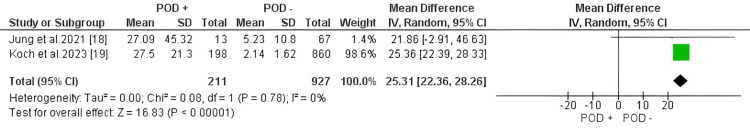
Forest plot of association of duration of intraoperative burst suppression with POD. POD: postoperative delirium

The pooled analysis of studies evaluating the duration of intraoperative burst suppression demonstrated a significant association with postoperative delirium (POD). Patients who developed POD had a markedly longer duration of burst suppression compared to those without POD, with a pooled mean difference of 25.31 min (95% CI: 22.36-28.26; p < 0.00001). Notably, heterogeneity was negligible (I² = 0%), indicating consistent findings across studies. These results strongly suggest that prolonged exposure to burst suppression during anesthesia is a significant risk factor for the development of postoperative delirium. Figure 4 presents the forest plot summarizing the pooled mean difference in duration of intraoperative burst suppression between POD and non-POD patients, indicating a significantly longer duration in those who developed delirium.

Individual Study Evidence

Fritz et al. established a clear dose-dependent relationship between EEG suppression duration and POD incidence (χ²{4} = 25, p < 0.0001) [16]. On multivariable analysis, each additional minute of intraoperative suppression independently increased delirium risk by 22% (adjusted OR: 1.22 per min; 99% CI: 1.06-1.40, p = 0.0002), demonstrating an additive effect of suppression duration. Koch et al. reinforced this relationship in the largest included cohort, showing that each additional minute of burst suppression increased POD risk by 1.1% (OR: 1.011; 95% CI: 1.000-1.022; p = 0.046) after adjustment for confounders [19]. Similarly, Jung et al. reported that patients who developed POD had more than five times longer suppression duration compared to non-delirium patients (27.09 ± 45.32 vs. 5.23 ± 10.80 min; p = 0.03), along with greater mean arterial pressure variability (p = 0.04) and deeper sevoflurane anesthesia (MAC: 1.22 ± 0.22 vs. 1.09 ± 0.17; p = 0.03), suggesting anesthetic depth as a shared upstream driver [18]. Lele et al. provided an important nuance, showing that although suppression was present in all POD-positive patients, the proportion of time in maximal suppression did not differ significantly (15.3% vs. 11.7%), indicating that the occurrence of any suppression may be more clinically relevant than its duration in TIVA-based protocols [20].

Pooled Odds Ratio for the Independent Association Between Burst Suppression and Postoperative Delirium

**Figure 5:**
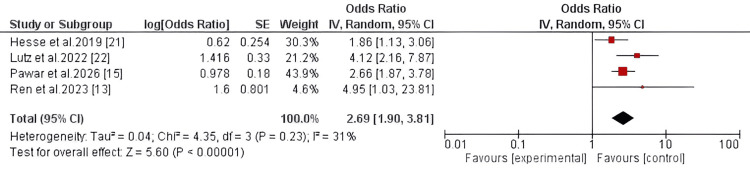
Forest plot of pooled odds ratio for the independent association between burst suppression and postoperative delirium.

The pooled analysis of four observational studies demonstrated a significant association between intraoperative EEG burst suppression and postoperative delirium in non-cardiac surgery patients. Using a random-effects model, the overall odds ratio was 2.69 (95% CI: 1.90-3.81; p < 0.00001), indicating that patients experiencing intraoperative burst suppression had approximately 2.7 times higher odds of developing postoperative delirium compared to those without burst suppression. All included studies showed a positive direction of effect, with individual odds ratios ranging from 1.86 to 4.95, although the magnitude of association varied. Heterogeneity among studies was low to moderate (I² = 31%, χ² = 4.35, p = 0.23), suggesting acceptable consistency in the observed effect across studies. Figure 5 depicts the forest plot of the pooled odds ratio for the independent association between intraoperative burst suppression and POD, confirming a consistent and significant increased risk across included observational studies.

Effect of Anesthetic Agent on Burst Suppression and Its Relationship to Postoperative Delirium

The choice of anesthetic agent was identified across multiple studies as an important determinant of suppression burden and, independently, of delirium risk. Koch et al. demonstrated that propofol-based general anesthesia was associated with the longest burst suppression durations (32.5 min), substantially exceeding those with sevoflurane (17.1 min) and desflurane (20.1 min) (p < 0.001) [19]. Notably, despite producing shorter suppression durations than propofol, desflurane was independently associated with a significantly elevated POD risk (OR: 1.766, 95% CI: 1.049-2.974, p = 0.032), even after controlling for suppression duration, indicating that anesthetic-specific neurotoxic mechanisms beyond burst suppression contribute to delirium risk with certain volatile agents. Jung et al. similarly demonstrated that patients who developed POD received significantly higher sevoflurane doses (MAC 1.22 ± 0.22 vs. 1.09 ± 0.17, p = 0.03), reinforcing the concept that anesthetic depth and agent selection are intertwined upstream modulators of both suppression burden and postoperative neurological outcomes [18]. Pawar et al. identified potential measurement discrepancies between visually scored and device-derived burst suppression ratios, cautioning that automated EEG-derived metrics may systematically underestimate the true clinical magnitude of suppression in some operative contexts [15].

Preoperative Cognitive Vulnerability and Frailty as Effect Modifiers

Multiple included studies identified preoperative cognitive function and frailty as clinically important effect modifiers of the burst suppression-POD relationship. Tang et al. demonstrated that patients with preoperative cognitive impairment accumulated greater intraoperative suppression and derived a disproportionately larger benefit from EEG-guided suppression reduction (interaction p = 0.01) [17]. Reese et al. confirmed that both burst suppression duration and pre-burst suppression EEG states were inversely associated with preoperative cognitive scores (β = -0.10 and -0.04 respectively, p < 0.05), with a moderate correlation between the two suppression-related EEG states (ρ = 0.52, p < 0.001), indicating that patients with lower baseline cognitive reserve are both more susceptible to intraoperative suppression and more likely to experience severe suppression events [14]. Ren et al. observed that frailty was significantly more prevalent among elderly patients who developed burst suppression during lumbar surgery (p = 0.048), further implicating diminished physiological and cognitive reserve as a key amplifier of suppression-related neurological risk in the non-cardiac operative setting [13].

Discussion

This systematic review and meta-analysis support a definitive reframing of intraoperative EEG burst suppression as follows: rather than an incidental marker of anesthetic depth, it constitutes a neurophysiologically meaningful risk factor for postoperative delirium (POD) in non-cardiac surgical patients. The convergence of multiple analytical approaches - binary occurrence, dose-dependent duration effects, and multivariable-adjusted estimates - across 10 studies and 3,574 patients yields a coherent and biologically plausible picture. The clinical implication is direct - burst suppression is modifiable, and its prevention during the maintenance phase of anesthesia represents a tangible target for reducing POD in high-risk surgical populations [13-22].

The observed pooled OR of 2.69 (95% CI: 1.90-3.81; p < 0.00001) is notably higher than estimates from prior meta-analyses that included mixed cardiac and non-cardiac populations. Park et al., which included 14 studies across mixed surgical populations including cardiac procedures, reported a lower pooled OR of 1.492 (95% CI: 1.022-2.178; I² = 44%) [8]. Similarly, Likhvantsev et al. observed a 41% relative increase in POD risk among patients with burst suppression (22.1% vs. 13.4%; p = 0.015) [9]. The larger effect size observed in the present analysis likely reflects its restriction to non-cardiac surgical populations. Cardiac surgery introduces additional delirium mechanisms, such as cardiopulmonary bypass-related microemboli, inflammatory cytokine release, and cerebral hypoperfusion, which act as independent risk factors and may dilute the specific contribution of burst suppression when pooled together. By excluding such procedures, this review isolates the burst suppression-delirium relationship in a setting where anesthetic exposure is the dominant modifiable factor, resulting in a more precise and etiologically coherent estimate. This is further supported by lower heterogeneity (I² = 31%) compared with Park et al. (I² = 44%) [8].

The dose-dependent relationship between burst suppression duration and POD risk is among the most compelling findings of this review, as it implies biological plausibility beyond simple association. Fritz et al., in a cohort of 619 patients, showed that each additional minute of intraoperative suppression increased delirium risk by 22% (OR: 1.22 per min; 99% CI: 1.06-1.40; p = 0.0002) [16]. Koch et al. reported a consistent incremental risk of 1.1% per min in the largest cohort (OR: 1.011; 95% CI: 1.000-1.022; p = 0.046) [19], while Jung et al. found markedly longer suppression durations in POD patients compared to non-delirious individuals (27.09 ± 45.32 vs. 5.23 ± 10.80 min; p = 0.03) [18]. While the I² of 0% in duration-based analyses should be interpreted with caution, given the small number of contributing studies, the directionality and magnitude of the effect are consistent across studies, lending credibility to a genuine dose-response phenomenon. However, an important nuance is highlighted by Lele et al., who observed in a TIVA-based spinal surgery cohort that although burst suppression occurred in all POD-positive patients, the proportion of time spent in maximal suppression did not differ significantly between groups (15.3% vs. 11.7%) [20]. This finding suggests the possibility of a threshold effect, particularly in propofol-based anesthesia, where the occurrence of any burst suppression may represent a critical neurological tipping point. In such contexts, prevention of any suppression, rather than modulation of its duration or intensity, may be the key clinical objective.

One of the most operationally actionable insights from this evidence base is that not all burst suppression carries equivalent delirium risk; rather, its temporal distribution within the anesthetic course is critically important. Lutz et al. demonstrated that maintenance-phase suppression was significantly associated with PACU delirium (52% vs. 20.9%; p = 0.002), whereas induction-phase suppression (48% vs. 61.5%; p = 0.223) and overall suppression prevalence (75% vs. 71.4%; p = 0.651) were not [22]. Pawar et al. corroborated these findings in the PANDA-G cohort, identifying maintenance-phase suppression as the more discriminating marker (67.2% vs. 46.3%; p = 0.004) [15]. Mechanistically, brief induction-phase suppression likely represents a transient electrocortical response to rapid loss of consciousness without sufficient duration or neurometabolic disruption to initiate the inflammatory and network-level cascades implicated in delirium pathogenesis [23]. In contrast, maintenance-phase suppression occurs during sustained surgical stress and the anesthetic steady state, when circulating stress mediators, including cortisol, interleukin-6, and tumor necrosis factor-alpha, and impaired cerebral autoregulation create a more vulnerable neurological milieu [24]. Jung et al. reinforced this interpretation, demonstrating that POD patients had higher sevoflurane concentrations during maintenance (MAC: 1.22 ± 0.22 vs. 1.09 ± 0.17; p = 0.03) and greater hemodynamic variability (p = 0.04) [18]. These findings suggest that excessive anesthetic depth and hemodynamic instability act synergistically to exacerbate suppression-related neurological injury during the maintenance phase. Accordingly, EEG monitoring vigilance and suppression-avoidance strategies should prioritize this intraoperative period.

The choice of anesthetic agent also emerges as an important modulator of suppression burden and delirium risk. Koch et al. reported that propofol-based anesthesia produced the longest suppression durations (32.5 min), compared with sevoflurane (17.1 min) and desflurane (20.1 min; p < 0.001) [19]. Despite shorter suppression durations than propofol, desflurane was independently associated with increased POD risk after adjustment (OR: 1.766; 95% CI: 1.049-2.974; p = 0.032), suggesting agent-specific neurotoxic or neuroinflammatory mechanisms beyond EEG suppression alone. This pharmacological dissociation is consistent with preclinical evidence suggesting that certain volatile agents, including desflurane, may exert direct neurotoxic effects via amyloid-beta oligomerization and tau phosphorylation pathways independent of depth of anesthesia [25]. This position treats burst suppression as an important component within a broader, agent-dependent pathophysiological framework rather than as the sole mediator of anesthetic neurotoxicity. Furthermore, Pawar et al. highlighted a key methodological limitation as follows: automated EEG indices may underestimate true suppression burden compared with visual scoring, introducing measurement error that may attenuate observed associations and complicate inter-study comparability [15].

Patient vulnerability significantly modifies the neurological impact of burst suppression. Tang et al., through the Anesthetic Depth and Postoperative Delirium Trial-2 (ADAPT-2) randomized trial, demonstrated that cognitively impaired patients accumulated greater suppression and derived a larger benefit from EEG-guided suppression reduction (interaction p = 0.01) [17]. Reese et al. further showed that lower preoperative cognitive scores were inversely associated with suppression duration and pre-burst EEG activity (β = -0.10 and -0.04; p < 0.05), indicating heightened susceptibility in patients with reduced cognitive reserve [14]. Ren et al. extended these findings to frailty, reporting an adjusted OR of 4.954 (95% CI: 1.034-23.736; p = 0.045) for POD among frail elderly patients undergoing spine surgery with burst suppression [13]. Similarly, Pawar et al. found that burst suppression was a stronger predictor of POD than age alone (β = 0.182; p = 0.002 vs. β = 0.009; p = 0.082) [15]. Collectively, these findings support the integration of preoperative cognitive and frailty assessments to guide intraoperative EEG monitoring intensity and thresholds.

Interventional evidence remains limited. The ADAPT-2 Trial successfully reduced intraoperative suppression exposure but did not demonstrate a statistically significant reduction in POD (17% vs. 20%; RR: 0.85; 95% CI: 0.47-1.5; p = 0.53) [17]. This likely reflects the multifactorial nature of delirium, where suppression avoidance alone is insufficient to offset risks from pain, sleep disruption, polypharmacy, and hemodynamic instability [14,16]. Additionally, the trial may have been underpowered to detect modest effect sizes. Future large-scale, multicomponent randomized trials targeting high-risk populations and multiple modifiable risk factors are needed to establish suppression-guided anesthesia as a preventive strategy.

Heterogeneity across studies was generally low to moderate, indicating reasonable consistency despite variability in design and populations. Differences in EEG monitoring methods, definitions of burst suppression (visual vs. processed indices), and delirium assessment tools (CAM, CAM-ICU, clinical diagnosis) contribute to residual variability [16,21]. Variations in statistical reporting, including adjusted vs. unadjusted estimates, may also influence pooled outcomes [13,15].

This review has several important limitations. The predominance of observational studies means residual confounding cannot be excluded despite multivariable adjustment. In addition, the review was not prospectively registered with PROSPERO, which reduced transparency and meant that subgroup analyses were interpreted as exploratory rather than confirmatory. The omission of Embase from the search strategy may have resulted in the omission of studies and introduced potential publication bias. Formal assessment of publication bias using funnel plots or Egger’s test was not possible because of the limited number of studies available for each meta-analysis. Outcome ascertainment was heterogeneous, with delirium assessed using validated tools such as the CAM and CAM-ICU, PACU-based clinical protocols, and trial-specific methods. Importantly, PACU delirium differs from ward-based postoperative delirium in timing, pathophysiology, and clinical significance; pooling these outcomes may have influenced the observed associations. The concentration of studies in elderly patients limits generalizability to younger surgical populations. Furthermore, the small number of studies reduced the precision of pooled estimates and restricted meaningful subgroup and sensitivity analyses. The pooled adjusted odds ratio should be considered a descriptive summary because studies adjusted for different confounders using different models. Likewise, I² = 0% should not be interpreted as proof of homogeneity. Finally, inconsistencies between automated and manual burst suppression measurements indicate non-uniform exposure definitions, limiting comparability and potentially attenuating true associations. Future research should prioritize adequately powered randomized trials, standardized quantification of suppression, mechanistic investigations, and multicomponent delirium-prevention strategies.

## Conclusions

In conclusion, this systematic review and meta-analysis demonstrate that intraoperative EEG burst suppression is significantly associated with an increased risk of postoperative delirium in patients undergoing non-cardiac surgery. Both the presence and duration of burst suppression show consistent relationships with POD, suggesting a dose-dependent effect, although variability exists across anesthetic techniques and patient populations. Importantly, factors such as preoperative cognitive impairment and frailty appear to amplify susceptibility to burst suppression and its adverse neurological consequences. While EEG-guided anesthetic strategies can reduce suppression exposure, their impact on delirium prevention remains uncertain, underscoring the multifactorial nature of POD. These findings support the role of burst suppression as a clinically relevant, potentially modifiable intraoperative marker and highlight the need for further high-quality randomized studies to clarify causality and optimize prevention strategies.
